# Sustained Aftereffect of α-tACS Lasts Up to 70 min after Stimulation

**DOI:** 10.3389/fnhum.2016.00245

**Published:** 2016-05-25

**Authors:** Florian H. Kasten, James Dowsett, Christoph S. Herrmann

**Affiliations:** ^1^Experimental Psychology Lab, Department of Psychology, European Medical School, Cluster for Excellence “Hearing for All”, Carl von Ossietzky UniversityOldenburg, Germany; ^2^German Center for Vertigo and Balance Disorders, Klinikum Grosshadern, Ludwig Maximilian University of MunichMunich, Germany; ^3^Research Center Neurosensory Science, Carl von Ossietzky UniversityOldenburg, Germany

**Keywords:** transcranial alternating current stimulation (tACS), transcranial electrical stimulation (TES), aftereffect, EEG, alpha oscillations

## Abstract

Transcranial alternating current stimulation (tACS) has been repeatedly demonstrated to increase power of endogenous brain oscillations in the range of the stimulated frequency after stimulation. In the alpha band this aftereffect has been shown to persist for at least 30 min. However, in most experiments the aftereffect exceeded the duration of the measurement. Thus, it remains unclear how the effect develops beyond these 30 min and when it decays. The current study aimed to extend existing findings by monitoring the physiological aftereffect of tACS in the alpha range for an extended period of 90 min post-stimulation. To this end participants received either 20 min of tACS or sham stimulation with intensities below their individual sensation threshold at the individual alpha frequency (IAF). Electroencephalogram (EEG) was acquired during 3 min before and 90 min after stimulation. Subjects performed a visual vigilance task during the whole measurement. While the enhanced power in the individual alpha band did not return back to pre-stimulation baseline in the stimulation group, the difference between stimulation and sham diminishes after 70 min due to a natural alpha increase of the sham group.

## Introduction

During the past decade transcranial alternating current stimulation (tACS) has emerged as a promising new method for non-invasive brain stimulation; several findings from human and animal research as well as neural network simulations provide evidence for its capability to entrain intrinsic brain oscillations via the application of sinusoidal currents on the scalp (i.e., Fröhlich and McCormick, 2010; Zaehle et al., 2010; Ali et al., 2013; Neuling et al., 2013; Helfrich et al., 2014b; Vossen et al., 2015; for a recent overview of human and animal findings see Herrmann et al., 2013; Reato et al., 2013). This feature makes tACS a promising technology to investigate causal relationships between neural oscillations and behavior or perception (Herrmann et al., 2013, 2015) as well as for the treatment of several neurological and psychiatric disorders in which dysfunctional neural oscillations are involved, such as Epilepsy, ADHD, Parkinson’s disease, Schizophrenia or Alzheimer’s disease (Herrmann and Demiralp, 2005; Uhlhaas and Singer, 2006, 2012; Brittain et al., 2013).

Besides behavioral (Antal et al., 2008; Laczó et al., 2012; Sela et al., 2012; Brignani et al., 2013; Strüber et al., 2014; Hoy et al., 2015; Vosskuhl et al., 2015) and physiological online effects of which the latter remain difficult to investigate (at least in humans) due to the massive artifact introduced to the signal (Helfrich et al., 2014b; Neuling et al., 2015; Witkowski et al., 2015) numerous studies demonstrated different types of physiological aftereffects following tACS application in various frequency bands and using different stimulation protocols (for a recent overview, see Veniero et al., 2015). For example Helfrich et al. (2014a) observed increased gamma-band coherence lasting for up to 20 min after applying either 20 min of in-phase or anti-phase gamma tACS targeting left and right extra-striate visual cortex. Wach et al. (2013) found a decrease in cortico-muscular coherence during isometric contraction in the gamma-band after tACS in the alpha band to persist for at least 38 min. Other studies demonstrated increased amplitudes of endogenous brain oscillations within the range of the stimulation frequency after tACS (Zaehle et al., 2010; Neuling et al., 2013; Vossen et al., 2015). However, Neuling et al. (2013) found this amplitude increase to be dependent on the current brain state during which tACS is administered; while an aftereffect was successfully produced during eyes-open (corresponding to low baseline alpha power), no increase in alpha power was observed under eyes-closed condition (accompanied by high baseline alpha power). A common finding of all these experiments was that the duration of the aftereffect exceeded the duration of the post stimulation measurement (up to 30 min). Thus, the development and duration of the tACS aftereffect beyond this point remains unclear. The current study aimed to extend existing findings on the time course of the tACS aftereffect. To this end the development of the aftereffect at the stimulated and neighboring frequency bands was monitored for a duration of 90 min following the application of 20 min tACS at participants’ individual alpha frequency (IAF). We hypothesized that power in the individual alpha band would increase in the stimulation group compared to both a control group receiving sham stimulation and to pre-stimulation baseline, at least during the first 30 min after tACS which would replicate previous findings (Neuling et al., 2013). However, during the following 60 min we expected the aftereffect to decay such that alpha power in the stimulation group no longer differs from sham or baseline alpha power.

## Materials and Methods

For comparability with previous findings the experimental procedures and data analysis in the current study follow the approaches of Zaehle et al. (2010) and Neuling et al. (2013) except for slight changes. The study was approved by the Ethics Committee of the University of Oldenburg and conducted in accordance with the Declaration of Helsinki.

### Participants

Twenty-two subjects participated in the experiment. All were students at the University of Oldenburg and received monetary compensation for participation and a performance dependent bonus (see “Paradigm” Section). Participants gave written informed consent prior to the experiment. They were medication-free at the day of the experiment and none of them reported presence or history of neurological or psychiatric disorders. All subjects were right-handed according to the Edinburgh handedness-scale (Oldfield, 1971). In a single-blind design participants were randomly assigned to one of the experimental groups (stimulation or sham) with the groups being counterbalanced for participants’ sex and time of measurement (sessions started either at 9 am or 2 pm). Subjects were debriefed immediately after the experiment. Due to technical issues the experiment had to be aborted for two subjects. A recent study reported tACS to be only effective with low baseline power in the targeted frequency band (Neuling et al., 2013). To avoid non-responsiveness to the stimulation due to such ceiling effects absolute baseline IAF power was *z*-transformed. Three participants exhibited *z*-scores exceeding 1.65 (corresponding to an α-level < 0.05, one-tailed) and were excluded from further analysis. Thus, 17 participants (stimulation group: 9, sham group: 8, age: 22.0 ± 2.24 years, 8 females) remained for analysis. An *a priori* power analysis based on the findings of Neuling et al. (2013) was conducted to estimate the required sample sizes. Results suggest sufficient power (1−β = 0.83) at a total sample size of 16 (eight per group). Therefore the obtained sample should be sufficient to detect effects of similar size. Furthermore, we provide effect sizes for all results as an additional measure independent of sample size.

### EEG

The Experiment was conducted in a dimly lit room with participants seated in a recliner in front of a computer screen at a distance of approximately 100 cm. The electroencephalogram (EEG) was measured from 10 sintered Ag-AgCl electrodes mounted in an elastic cap (EasyCap GmbH, Herrsching, Germany) placed at five frontal and five parietal positions around Fz and Pz following the international 10–20 system layout. An electrode attached to the nose served as reference. The ground electrode was positioned at Fpz. Additionally a vertical Electrooculogram (EOG) was recorded underneath the right eye to monitor eye-movements during the experiment. All impedances were kept below 10 kΩ. EEG was recorded using a BrainAmp (Brain Products GmbH, Gilching, Germany) amplifier and the BrainVision Recorder Software (Brain Products GmbH, Gilching, Germany). Data were sampled at a rate of 250 Hz and a resolution of 0.5 μV to increase the voltage range of the amplifier avoiding clipping effects during tACS application. A DC reset was applied when the amplifier ran into saturation.

Prior to the main experiment participants IAF was determined by 90 s of eyes-closed resting EEG. The obtained EEG data were segmented into 1 s epochs. Subsequently a Fast Fourier Transform (FFT) was applied to each epoch to compute power spectra. The first 50 artifact free spectra were averaged and the power peak in the 8–12 Hz range at electrode Pz was visually identified and used as stimulation frequency for the main experiment. If no clear peak was evident the procedure was repeated.

**Figure 1:**
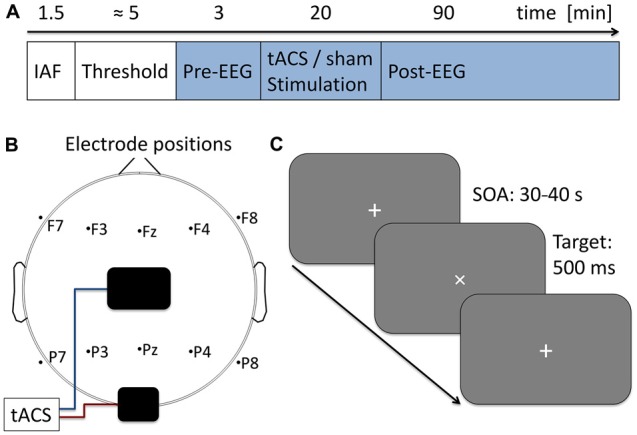
**Experimental procedure. (A)** Time course of the current experiment. First 90 s of eyes-closed EEG were acquired to determine participants’ individual alpha frequency (IAF) which was used as stimulation frequency in the subsequent steps. Next, stimulation intensity was adjusted to the individual sensation threshold. During the following 113 min participants performed a visual vigilance task (indicated in blue) while 3 min of baseline EEG was measured followed by 20 min of tACS or sham stimulation and 90 min post-stimulation EEG. **(B)** Electrode setup. Stimulation electrodes were placed above Cz (5 × 7 cm) and Oz (4 × 4 cm) following the international 10–20 system. Additionally 10 EEG electrodes were positioned over five frontal and five parietal sides. **(C)** Visual vigilance task. Participants fixated a white cross at the center of a computer screen. Every 30–40 s the cross was rotated by 45° for a duration of 500 ms. Participants were given 2 s to manually respond to the rotation and received 0.05 € for each detected target. A total of 191 targets were presented during the experiment.

EEG was recorded during the whole course of the main experiment. In the beginning 3 min of baseline EEG were obtained, followed by 20 min of tACS or sham stimulation. Subsequently another 90 min of post-tACS EEG were acquired (for an overview of the time course of the experiment, see Figure 1A).

### Electrical Stimulation

Stimulation was administered by two surface conductive rubber electrodes attached to participants’ scalp. One was positioned centered above Cz (5 × 7 cm), the other above Oz (4 × 4 cm) using an adhesive, electrically conductive paste (ten20 conductive paste, Weaver and Co., USA). In a recent modeling study this montage has been shown to produce highest current densities in posterior brain regions (Neuling et al., 2012). A smaller electrode over Oz was used to further increase current density in occipital areas below the electrode. An overview of the EEG and tACS montage is given in Figure 1B. Electrodes were connected to a battery-operated stimulator system (DC Stimulator Plus, Neuroconn, Ilmenau, Germany). The stimulation signal was digitally sampled at 100 kHz using Matlab 2012a (The MathWorks Inc., Natick, MA, USA) and sent in chunks of 1 s segments to a digital to analog converter (Ni USB 6229, National Instruments, Austin, Texas, USA) converting the digital sinusoidal signal into an analog output for the stimulator. Electrode impedance was kept below 10 kΩ. Participants were stimulated at their IAF. Intensity of the stimulation was adjusted to subjects’ individual sensation threshold which was defined as the highest intensity at which participants did not notice the stimulation (no phosphene or skin sensation). To determine the threshold participants were stimulated with an initial intensity of 1000 μA (peak-to-peak). If participants noticed the stimulation, intensity was decreased in steps of 100 μA until they did not notice the stimulation anymore. In case participants did not notice the initial stimulation, intensity was increased in steps of 100 μA until they noticed the stimulation. Each of the steps was applied for 20 s, without fade-in or fade-out. The obtained intensity was used as the stimulation intensity during the experiment. On average stimulation intensity was 1200 μA (± 440 μA, min: 400 μA, max: 1800 μA) peak-to-peak with an average frequency of 10 Hz (±1.12 Hz). Student’s two-sample *t*-test revealed no significant difference in intensities (*t*_15_ = −0.22, *p* = 0.83, *d* = 0.1) or stimulation frequencies (*t*_15_ = −0.42 *p* = 0.68, *d* = 0.2) between experimental groups. After 3 min of baseline EEG the experimental group received 20 min of tACS with 10 s fade-in and fade-out at the beginning and the end of the stimulation period (intensity was increased/decreased every second by 1/10 of the final stimulation intensity). While all other stimulation parameters were kept the same as in the experimental group the sham group received only 30 s of stimulation (including 10 s fade-in and fade-out) in the beginning of the 20 min period.

### Paradigm

To ensure participants being awake and attentive they performed a visual vigilance task during the whole course of the main experiment (baseline, tACS, post-tACS measurement). Visual stimuli were delivered simultaneously with the tACS signal generation using Matlab and the Psychtoolbox 3. Stimuli were displayed on a computer screen (Samsung SyncMaster P2470H, 1920 × 1080 pixels, 60 Hz refresh rate) at a distance of approximately 100 cm. Subjects were instructed to fixate a white cross (diameter 1.58°) at the center of the screen which was rotated by 45° for 500 ms every 30–40 s. Participants had to manually respond to each of the rotations within 2 s after stimulus onset (see Figure 1C). To maintain subjects motivation they received a bonus of 0.05 € for each hit. A total of 191 targets were presented during the 113 min of the experiment.

### Debriefing

After finishing the experiment participants were asked to fill out a translated version of an adverse effects questionnaire introduced by Brunoni et al. (2011). The questionnaire assesses the 10 most commonly reported adverse effects during transcranial electric stimulation (headache, neck pain, scalp pain, tingling, itching, burning sensation, skin redness, sleepiness, trouble concentrating and acute mood change). Subjects had to rate the intensity of each adverse effect (1 – none, 2 – mild, 3 – moderate, 4 – severe) and how strongly they attributed them to tACS (1 – none, 2 – remote, 3 – probable, 4 – definite). To confirm participants’ blindness towards their experimental condition they were finally asked to guess whether they had been stimulated or not. Immediately afterwards they were informed about their true experimental condition and the aims of the study.

### Data Analysis

Data analysis was performed using Matlab 2012b and the Fieldtrip toolbox (Oostenveld et al., 2011). For statistical analysis statistical software R 3.2.3 (R Foundation for Statistical Computing, Vienna, Austria) was used.

EEG data were high-pass filtered at 0.3 Hz, low-pass filtered at 100 Hz and subsequently segmented into 3 min blocks resulting in one baseline block prior to and 30 blocks after tACS. EEG data acquired during tACS application were not further analyzed. Each block was subsequently divided into 180 non-overlapping 1 s epochs. Segments containing visual stimulation or manual responses were removed as well as epochs containing artifacts. FFT spectra (Hanning window, 2 s zero-padding) were computed and averaged for the first 120 artifact free epochs in each 3 min block. From these spectra, power in the individual alpha band (IAF ± 2 Hz) was obtained and averaged for each block. To account for inter-individual differences, IAF band power in the post stimulation blocks was normalized with respect to pre-stimulation baseline. Data for three subsequent 30 min time periods were analyzed separately using three rmANOVAs to ensure comparability with the results of Neuling et al. (2013) and to preserve the opportunity of assumption testing which is only possible with more observations than levels of measurement. Each rmANOVA was conducted with the within subject factor *time* (10 levels) and the between subject factor *group* (two levels, *stimulation* vs. *sham*). Please note that due to the previous normalization only post stimulation data were analyzed and a stimulation effect would therefore reveal itself as a significant main effect of the factor *group*. Separate two-sided *t*-tests for stimulation and sham group against baseline were computed to test for deviations from baseline IAF band power for each of the conditions. All obtained *p*-values were Bonferroni-corrected to account for multiple comparisons. Greenhouse-Geisser corrected values are reported in case sphericity was violated. Furthermore, power in an upper (IAF + 3 Hz to IAF + 5 Hz) and a lower frequency band (IAF − 5 Hz to IAF − 3 Hz) were analyzed with the same procedure to ensure frequency specificity of the tACS effect. Finally, a set of FDR corrected, one-sided *post hoc t*-tests on relative IAF band power between stimulation and sham group were calculated for each of the 3 min post-tACS blocks to determine the point in time were the tACS aftereffect vanishes.

Statistical analysis of participants’ ratings on adverse effects was performed using Wilcoxon rank sum test for independent samples. To improve chances to detect undesired group differences no *p*-value correction was applied. Participants guesses about their assigned experimental condition was analyzed using Fisher’s exact test for count data.

## Results

### Debriefing

The most reported adverse effects (intensities rated higher than 1) after the experiment were *sleepiness* (82.35%), *trouble concentrating* (64.70%) and *tingling* (41.17%). Ratings for intensity of adverse effects were generally relatively low, except for *sleepiness* (*M* = 2.71) and *trouble concentrating* (*M* = 2.12). For the ratings on whether subjects attributed the adverse effects to the stimulation only *tingling* achieved an average score above 2 (remote, *M* = 2.18). Two-sided *t*-test between experimental groups revealed no significant differences between groups for any of the items (all *p* > 0.08). After filling out the questionnaire 41.2% of subjects indicated that they thought they were stimulated during the experiment (33.3% in the *stimulation* group 50% in the *sham* group). Fisher’s exact test for count data confirmed that participants were unaware of their experimental condition (*OR* = 0.52, *p* = 0.63).

### Vigilance Task

On average participants detected 96.61% (± 6.01%) of all targets. None of them performed worse than 80%, confirming that participants were vigilant and attentive during the experiment. A Students’ two sample *t*-test revealed no difference between experimental groups (*M_stim_* = 97.61, *M_sham_* = 95.48; *t*_15_ = 0.72, *p* = 0.48, *d* = 0.35).

### Electrophysiological Data

**Figure 2:**
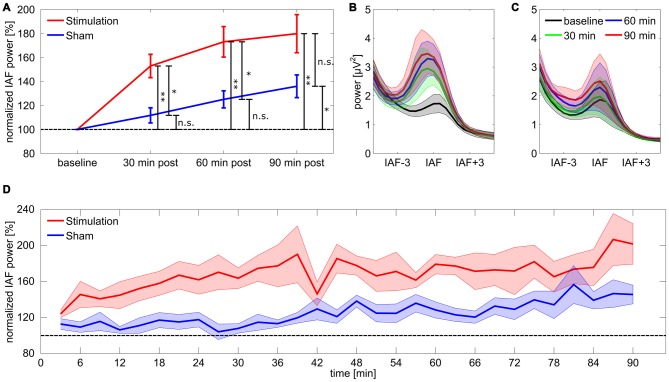
**Power change in the individual alpha band. (A)** Post period increase in the individual alpha band. Stars coding for significant differences (* < 0.05, ** < 0.01). Error bars reflect standard error of the mean (SEM), the dashed line reflects baseline level. **(B,C)** Spectra for stimulation **(B)** and sham **(C)** group aligned on IAF and averaged over subjects. **(D)** Time course of normalized power in the individual alpha band for stimulation and sham group. Shaded areas around the lines depict SEM, the dashed line reflects baseline level.

For the 17 subjects included in the final analysis the rmANOVA on relative IAF band power for the first 30 min post-tACS revealed a significant main effect of *group* (*F*_(1,15)_ = 11.88, *p* = 0.011, *η^2^* = 0.3), but no effect of *time* (*F*_(9,135)_ = 1.75, *p* = 0.44, *η^2^* = 0.05) or a *group × time* interaction (*F*_(9,135)_ = 1.78, *p* = 0.42, *η^2^* = 0.05). Subsequent Bonferroni-corrected *post hoc t*-tests against baseline showed a significant divergence from baseline for the *stimulation* group (*t*_8_ = 5.43, *p* = 0.004, *d* = 1.8) but not for *sham* (*t*_7_ = 1.86, *p* = 0.62, *d* = 0.66). Results demonstrate that power in the IAF band was increased in the *stimulation* group compared to *sham* and to baseline, while power in the *sham* group remained at baseline level. The rmANOVA for the second 30 min post-tACS shows a similar pattern with a significant main effect of *group* (*F*_(1,15)_ = 10.12, *p* = 0.019, *η^2^* = 0.26) but neither an effect of *time* (*F*_(9,135)_ = 0.70, *p* = 1, *η^2^* = 0.02) nor a significant *group × time* interaction (*F*_(9,135)_ = 1.36, *p* = 0.78, *η^2^* = 0.04). *Post hoc t*-test exhibited a significant deviation from baseline for the *stimulation* group (*t*_8_ = 5.75, *p* = 0.003, *d* = 1.9) but not for *sham* (*t*_7_ = 3.53, *p* = 0.058, *d* = 1.2) suggesting that power in the IAF band remains increased in the *stimulation* group compared to baseline and to *sham*. However, for the last 30 min period the rmANOVA revealed neither a significant effect of *group* (*F*_(1,15)_ = 4.75, *p* = 0.14, *η^2^* = 0.17) nor an effect of *time* (*F*_(9,135)_ = 1.96, *p* = 0.32, *η^2^* = 0.04) or a significant *group × time* interaction (*F*_(9,135)_ = 0.72, *p* = 1, *η^2^* = 0.02). *Post hoc t*-tests suggest a significant difference from baseline IAF band power for both *stimulation* (*t*_8_ = 4.85, *p* = 0.007, *d* = 1.61) and *sham* (*t*_7_ = 3.75, *p* = 0.04, *d* = 1.2). Results suggest that the difference in IAF band power between *stimulation* and sham *group* vanishes, due to power increase in the IAF band in the sham group (refer to Figure 2 for an overview).

**Figure 3:**
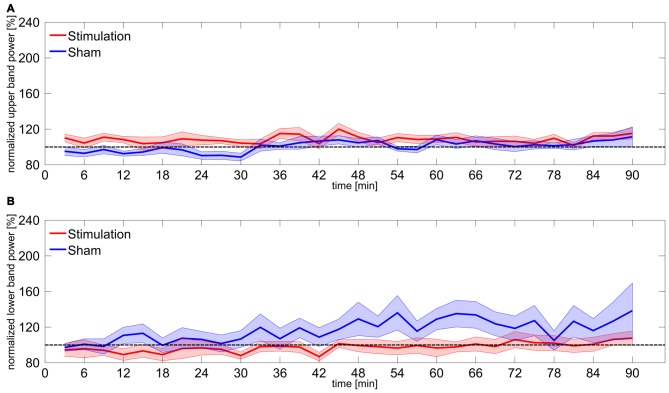
**Power change in upper and lower frequency bands. (A)** Time course of normalized power in the upper frequency band 3–5 Hz above participants IAF. Shaded areas around the lines reflect SEM, dashed line reflects baseline level. **(B)** Time course of normalized power in the lower frequency band 3–5 Hz below participants IAF. Shaded areas around the lines reflect SEM, dashed line reflects baseline level.

Statistical analysis of the upper and lower frequency bands revealed no significant effects. However, the rmANOVA on upper band power during the first 30 min shows a marginal effect of *group* (*F*_(1,15)_ = 6.72, *p* = 0.06, *η^2^* = 0.18). Bonferroni-corrected *t*-tests against baseline confirmed that neither of the groups significantly differed from baseline (*stimulation*: *t*_8_ = 1.77, *p* = 0.69, *d* = 0.59; *sham*: *t*_7_ = −2.05, *p* = 0.48, *d* = 0.72). A detailed overview on results of upper and lower band power is given in Table 1. Time courses of upper and lower band power are illustrated in Figure 3.

**Table 1:** **Results for upper and lower band**.

rmANOVA	*F*	*p*	*η^2^*	*t*-test vs. baseline	*T*	*p*	*d*
Lower first 30 min
*Group*	1.30	0.81	0.06	*Stim*	1.06	1.00	0.35
*Time*	0.68	1.00	0.01
*Group × time*	1.02	1.00	0.02	*Sham*	0.58	1.00	0.21
Lower second 30 min
*Group*	3.38	0.26	0.14	*Stim*	0.47	1.00	0.16
*Time*	1.57	0.58	0.03
*Group × time*	1.16	1.00	0.02	*Sham*	1.75	0.74	0.62
Lower third 30 min
*Group*	1.94	0.55	0.09	*Stim*	0.31	1.00	0.10
*Time*	1.34	0.82	0.02
*Group × time*	1.37	0.80	0.02	*Sham*	1.60	0.93	0.56
Upper first 30 min
*Group*	6.72	0.06^T^	0.18	*Stim*	1.77	0.69	0.59
*Time*	0.77	1.00	0.03
*Group × time*	0.45	1.00	0.01	*Sham*	2.05	0.48	0.72
Upper second 30 min
*Group*	1.60	0.68	0.05	*Stim*	2.98	0.01	0.99
*Time*	1.44	0.53	0.05
*Group × time*	1.24	0.84	0.04	*Sham*	1.01	1.00	0.35
Upper third 30 min
*Group*	0.45	1.00	0.02	*Stim*	2.50	0.22	0.83
*Time*	1.49	0.65	0.05
*Group × time*	0.31	1.00	>0.01	*Sham*	1.01	1.00	0.35

*rmANOVA and t-test results for normalized power in the lower (IAF − 3 Hz to IAF − 5 Hz) and upper (IAF + 3 Hz to IAF + 5 Hz) frequency bands. Analysis follows the same procedure as for the normalized IAF data. Left half shows ANOVA results, right half results for the comparisons of each group against baseline. None of the analysis exhibited significant results. Only a trend for the factor group is evident in the upper band during the first 30 min (indicated by upper case T)*.

**Figure 4:**
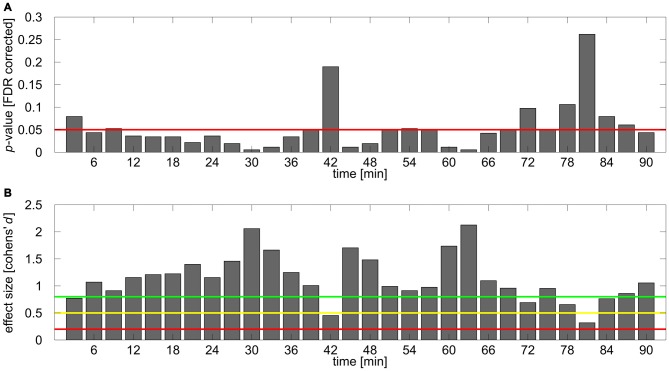
**Results of *post hoc* analysis. (A)** FDR corrected *p*-values for the comparison of normalized IAF band power between stimulation and sham group for each time bin. Red line depicts 0.05 significance boundary. **(B)** Corresponding effect sizes (Cohen’s *d*) for each of the comparisons. Colored lines depict suggestions for small (*d* = 0.2; red line), medium (*d* = 0.5; yellow line) and large (*d* = 0.8, green line) effects given by Cohen (1992).

To further investigate the time course of the tACS aftereffect a set of 30 one-sided FDR-corrected *t*-tests comparing relative alpha power between *stimulation* and *sham* group were calculated for each of the 3 min blocks. The obtained *p*-values are illustrated in Figure 4A. The corresponding effect sizes (Cohen’s *d*) are shown in Figure 4B. Most of the comparisons yielded significant or very close to significant differences between groups, however during the first 20 comparisons time bin 1 (0–3 min post-tACS) and 14 (39–42 min post-tACS) clearly failed to reach significance. After around 70 min several comparisons exhibit non-significant results supporting the corresponding ANOVA results by showing that the aftereffect begins to vanish around this time period.

## Discussion

The current study aimed to elucidate the time course and duration of the tACS aftereffect in the alpha band beyond 30 min after stimulation. Results successfully replicate the aftereffect reported by Neuling et al. (2013) during the first 30 min after tACS and demonstrate the group difference between stimulation and sham group to persist up to 70 min. However, this diminishing group effect is due to a natural alpha rise in the sham group rather than a decrease of alpha power back to baseline level in the stimulation group. The findings are in line with studies investigating electrophysiological correlates of vigilance, time on task and mental fatigue reporting an increase in alpha power over time, especially at occipital and parietal electrode sides (Daniel, 1967; Cajochen et al., 1995; Boksem et al., 2005; Oken et al., 2006). In summary, these results suggest alpha power is unlikely to fall back to baseline for neither stimulation nor sham group during any of the commonly used vigilance paradigms which have been used to investigate the aftereffect in the alpha band (Zaehle et al., 2010; Neuling et al., 2013; Vossen et al., 2015). This emphasizes the importance of carefully chosen criteria for the definition of the aftereffect which can be either compared to its own pre stimulation baseline or to a sham condition. In the case of alpha band stimulation it is more reasonable to define the aftereffect as the difference between stimulation and sham group instead of the difference to a pre-stimulation baseline since the latter does not account for participants’ natural alpha increase.

By comparing stimulation and sham group in smaller time bins the current study tried to reveal further insights into the time course of the stimulation aftereffect. The effect appears to build up during the first minutes of the post-tACS measurement and stabilizes afterwards. Espeacially in the first time bin which samples alpha power within the first 3 min after tACS the aftereffect appears to be relatively weak, if present at all (Figures 2D, 4). A similar pattern can also be found in the data of Neuling et al. (2013) but has neither been analyzed nor described in more detail there since the time course of alpha power in the stimulation group was only tested against baseline and not compared to the corresponding time course of the sham group. This observation provides further support for the idea that on- and offline effects of tACS reflect distinct processes (Veniero et al., 2015; Vossen et al., 2015). While attempts to measure the online effects of tACS in humans and animal data suggest entrainment as the core underlying mechanism during tACS (Fröhlich and McCormick, 2010; Helfrich et al., 2014b; Neuling et al., 2015; Witkowski et al., 2015), data from offline measurements and neural-network simulations favor mechanisms of synaptic plasticity, e.g., spike timing dependent placticity, to account for aftereffects (Zaehle et al., 2010; Neuling et al., 2013; Veniero et al., 2015; Vossen et al., 2015). On the other hand, there is some evidence which suggests that online and aftereffects are not completely indepent. For example Helfrich et al. (2014a,b) demonstrated correlations between the strength of online entrainment with aftereffect strength. It seems plausible to assume that an online effect of entrainment is necessary before an offline effect of synaptic plasticity can be observed. Further insights into the underlying physiological procesess during and after tACS could be achieved by adapting the approach of Nitsche et al. (2003a). By selectively blocking sodium and calcium channels as well as NMDA receptors by pharmacological treatment they were able to demonstrate the involvement of calcium and sodium channels in the generation of online- and aftereffects of anodal transcranial direct current stimulation (tDCS) but not of cathodal tDCS as well as an additional involvement of NMDA receptors in the generation of both cathodal and anodal tDCS aftereffects. A similar role of NMDA receptors after tACS application would be particularly interesting because these receptors are involved in procesees of synaptic placticity such as long-term potentiation and long-term depression (Bennett, 2000; Nitsche et al., 2003a; Lüscher and Malenka, 2012).

Interestingly, within the time bin 39–42 min after stimulation the aftereffect in the current study appears to collapse and immedeately build up again. From the data at hand it remains unclear which mechanism accounts for this phenomenon or whether it is a random effect resulting from participants waxing and waning in alpha power. However, single subject time courses in the stimulation group quite consistently exhibit negative slopes around this time bin. During the subsequent minutes the effect builds up again until it begins to vanish after around 70 min as indicated by several subsequent *t*-tests failing to reach significance. This duration falls approximately in the same range as aftereffects reported for tDCS evaluated by means of motor-evoked-potentials, which last up to 60 min for cathodal tDCS (Nitsche et al., 2003b) and up to 90 min for anodal tDCS (Nitsche and Paulus, 2001).

The current study provides first evidence for the development and total duration of the tACS aftereffect in the alpha band. However, the results can only provide a first step towards understanding the dynamics and long term effects of tACS. For example, it remains unclear how far stimulation parameters like stimulation duration, intensity and matching between stimulation and individual peak frequency in the targeted frequency band affect duration and amplitude of the aftereffect. Two recent studies pointed out the importance of stimulation duration for the successful production of an aftereffect (Strüber et al., 2015; Vossen et al., 2015). Furthermore, one of the studies found correlations between the mismatch between stimulation and individual peak frequency and aftereffect (Vossen et al., 2015). But so far these relationships have not been systematically investigated. For tDCS an almost linear relationship between stimulation duration and aftereffect duration has been demonstrated (Nitsche and Paulus, 2001; Nitsche et al., 2003b) as well as an increase of aftereffect strength with stimulation amplitude (Nitsche and Paulus, 2000). Additionally, some authors emphasized the role of the brain state during which tACS is applied (Neuling et al., 2013; Kar and Krekelberg, 2014; Kar, 2015). According to these authors stimulation is only effective in modulating behavior and physiology when applied during a brain state involving the stimulated frequency band. These aspects are crucial since deviations in stimulation parameters as compared to the ones used in the current study, especially weaker intensities or shorter durations, might lead to weaker and/or shorter aftereffects or, in the worst case, to no effect at all. On the other hand, despite the vanishing difference between stimulation and sham group 70 min after stimulation in the current results, it cannot be ruled out that plastic changes induced by tACS might persist on even larger scales of hours or even days. Long term measurements including several measurements for example within the course of a week could shed light on this question.

## Author Contribtutions

FHK, JD, CSH: designed the study; FHK: acquired the data; FHK, JD: analyzed the data; FHK, JD, CSH: wrote the article.

## Conflict of Interest Statement

The authors declare that the research was conducted in the absence of any commercial or financial relationships that could be construed as a potential conflict of interest.
